# Inside the Mucosal Immune System

**DOI:** 10.1371/journal.pbio.1001397

**Published:** 2012-09-25

**Authors:** Jerry R. McGhee, Kohtaro Fujihashi

**Affiliations:** Department of Pediatric Dentistry, The School of Dentistry, The University of Alabama at Birmingham, Birmingham, Alabama, United States of America

## Abstract

An intricate network of innate and immune cells and their derived mediators function in unison to protect us from toxic elements and infectious microbial diseases that are encountered in our environment. This vast network operates efficiently by use of a single cell epithelium in, for example, the gastrointestinal (GI) and upper respiratory (UR) tracts, fortified by adjoining cells and lymphoid tissues that protect its integrity. Perturbations certainly occur, sometimes resulting in inflammatory diseases or infections that can be debilitating and life threatening. For example, allergies in the eyes, skin, nose, and the UR or digestive tracts are common. Likewise, genetic background and environmental microbial encounters can lead to inflammatory bowel diseases (IBDs). This mucosal immune system (MIS) in both health and disease is currently under intense investigation worldwide by scientists with diverse expertise and interests. Despite this activity, there are numerous questions remaining that will require detailed answers in order to use the MIS to our advantage. In this issue of *PLOS Biology*, a research article describes a multi-scale in vivo systems approach to determine precisely how the gut epithelium responds to an inflammatory cytokine, tumor necrosis factor-alpha (TNF-α), given by the intravenous route. This article reveals a previously unknown pathway in which several cell types and their secreted mediators work in unison to prevent epithelial cell death in the mouse small intestine. The results of this interesting study illustrate how in vivo systems biology approaches can be used to unravel the complex mechanisms used to protect the host from its environment.

Higher mammals have evolved a unique mucosal immune system (MIS) in order to protect the vast surfaces bathed by external secretions (which may exceed 300 m^2^ in humans) that are exposed to a rather harsh environment. The first view of the MIS is a single-layer epithelium covered by mucus and antimicrobial products and fortified by both innate and adaptive components of host defense (Figure 1). To this, we can add a natural microbiota that lives in different niches, i.e., the distal small intestine and colon, the skin, the nasal and oral cavities, and the female reproductive tract. The largest microbial population can reach ∼10^12^ bacteria/cm^3^ and occurs in the human large intestine [1]–[3]. This large intestinal microbiota includes over 1,000 bacterial species and the individual composition varies from person-to-person. Other epithelial sites harbor a separate type of microbiota, including the mouth, nose, skin, and other wet mucosal surfaces, that contributes to the host; in turn, the host benefits its microbial co-inhabitants. Gut bacteria grow by digesting complex carbohydrates, proteins, vitamins, and other components for absorption by the host, which in return rewards the microbiota by developing a natural immunity and tolerance (reviewed in [4]–[7]). Finally, the host microbiota influences the development and maturation of cells within lymphoid tissues of the MIS [8],[9].

**Figure 1:**
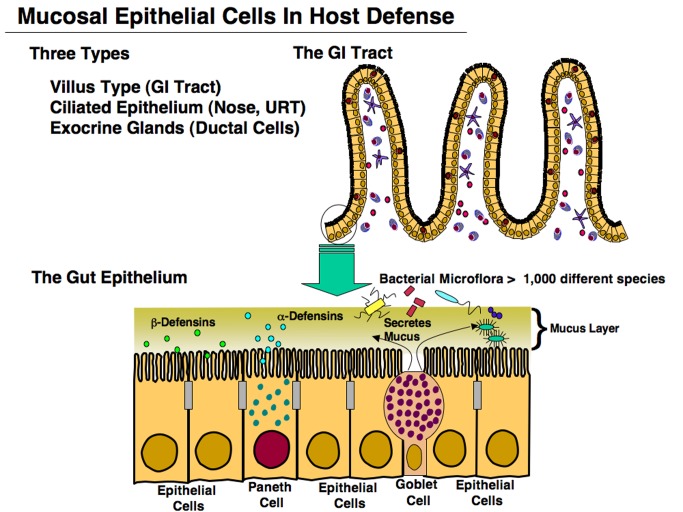
The gut, nasal, upper respiratory and salivary, mammary, lacrimal, and other glands consist of a single layered epithelium. Projections of villi in the GI tract consist mainly of columnar epithelial cells (ECs), with other types including goblet and Paneth cells. Goblet cells exhibit several functions including secretion of mucins, which form a thick mucus covering. Paneth cells secrete chemokines, cytokines, and anti-microbial peptides (AMPs) termed α-defensins.

Mucosal epithelial cells (ECs) are of central importance in host defense by providing both a physical barrier and innate immunity. For example, goblet cells secrete mucus, which forms a dense, protective covering for the entire epithelium (Figure 1). Peristalsis initiated by the brush border of gastrointestinal (GI) tract ECs allows food contents to be continuously digested and absorbed as it passes through the gut. In the upper respiratory (UR) tract, ciliated ECs capture inhaled, potentially toxic particles, and their beating moves them upward to expel them, thereby protecting the lungs. Damaged, infected, or apoptotic ECs in the GI tract move to the tips of villi and are excreted; newly formed ECs arise in the crypt region and continuously migrate upward. Paneth cells in crypt regions of the GI tract produce anti-microbial peptides (AMPs), or α-defensins, while ECs produce β-defensins [10],[11] for host protection (Figure 1). A major resident cell component of the mucosal epithelium are intraepithelial lymphocytes (IELs). The IELs consist of various T cell subsets that interact with ECs in order to maintain normal homeostasis [12]. Regulation is bi-directional, since ECs can also influence IEL T cell development and function [12]–[14].

The MIS, simply speaking, can be separated into inductive and effector sites based upon their anatomical and functional properties. The migration of immune cells from mucosal inductive to effector tissues via the lymphatic system is the cellular basis for the immune response in the GI, the UR, and female reproductive tracts (Figure 2). Mucosal inductive sites include the gut-associated lymphoid tissues (GALT) and nasopharyngeal-associated lymphoid tissues (NALT), as well as less well characterized lymphoid sites (Box 1). Collectively, these comprise a mucosa-associated lymphoid tissue (MALT) network for the provision of a continuous source of memory B and T cells that then move to mucosal effector sites 13,14. The MALT contains T cell regions, B cell–enriched areas harboring a high frequency of surface IgA-positive (sIgA^+^) B cells, and a subepithelial area with antigen-presenting cells (APCs), including dendritic cells (DCs) for the initiation of specific immune responses (Figure 2). The MALT is covered by a subset of differentiated microfold (M) cells, ECs, but not goblet cells, and underlying lymphoid cells that play central roles in the initiation of mucosal immune responses. M cells take up antigens (Ags) from the lumen of the intestinal and nasal mucosa and transport them to the underlying DCs (Figure 2). The DCs carry Ags into the inductive sites of the Peyer's patch or via draining lymphatics into the mesenteric lymph nodes (MLNs) for initiation of mucosal T and B cell responses (Figure 2). Retinoic acid (RA) producing DCs enhance the expression of mucosal homing receptors (α_4_β_7_ and CCR9) on activated T cells for subsequent migration through the lymphatics, the bloodstream, and into the GI tract lamina propria [15],[16]. Regulation within the MIS is critical; several T cell subsets including Th1, Th2, Th17, and Tregs serve this purpose [13],[14],[17] (Figure 2).

**Box 1. Major Inductive Sites for Mucosal Immune Responses**

- GALT (gut-associated lymphoid tissues)
  - Peyer's patches (PPs)
  - Mesenteric lymph nodes (MLNs)
  - Isolated lymphoid follicles (ILFs)
- NALT (nasopharyngeal-associated lymphoid tissues)
  - Tonsils/adenoids
  - Inducible bronchus-associated lymphoid tissue (iBALT)
  - Cervical lymph nodes (CLNs)
  - Hilar lymph nodes (HLNs)

**Figure 2:**
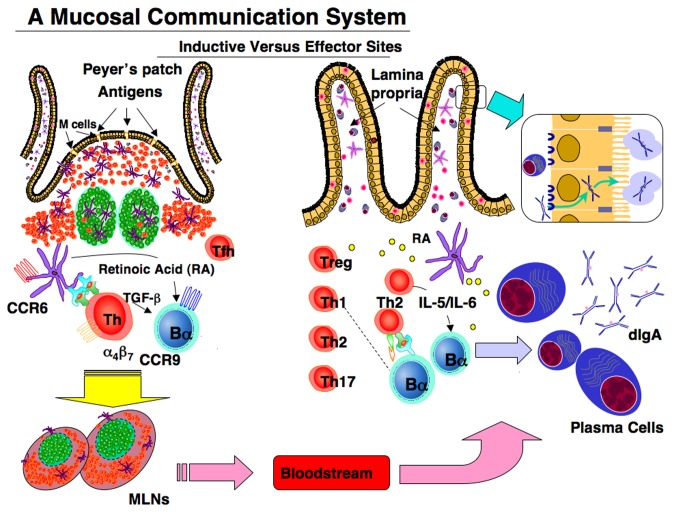
The mucosal immune system (MIS) is interconnected, enabling it to protect vast surface areas. This is accomplished by inductive sites of organized lymphoid tissues, e.g., in the gut the Peyer's patches (PPs) and mesenteric lymph nodes (MLNs) comprise the GALT. Lumenal Ags can be easily sampled via M cells or by epithelial DCs since this surface is not covered by mucus due to an absence of goblet cells. Engested Ags in DCs trigger specific T and B cell responses in Peyer's patches and MLNs. Homing of lymphocytes expressing specific receptors helps guide their eventual entry into major effector tissues, e.g., the lamina propria of the gut, the upper respiratory (UR) tract, the female reproductive tract, or acinar regions of exocrine glands. Terminal differentiation of plasma cells producing polymeric (mainly dimeric) IgA is then transported across ECs via the pIgR for subsequent release as S-IgA Abs.

Mucosal effector sites, including the lamina propria regions of the GI, the UR and female reproductive tracts as well as secretory glandular tissues (i.e., mammary, lacrimal, salivary, etc.) contain Ag-specific mucosal effector cells such as IgA-producing plasma cells, and memory B and T cells [18]. Adaptive mucosal immune responses result from CD4^+^ T cell help (provided by both CD4^+^ Th2 or CD4^+^ Th1 cells), which supports the development of IgA-producing plasma cells (Figure 2). Again, the ECs become a central player in the MIS by producing the polymeric Ig receptor (pIgR) (which binds both polymeric IgA and IgM) [19]. Lamina propria pIgA binds the pIgR on the basal surface of ECs, the bound pIgA is internalized, and then transported apically across the ECs (Figure 2). Release of pIgA bound to a portion of pIgR gives rise to secretory IgA (S-IgA) antibodies (Abs) with specificities for various Ags encountered in mucosal inductive sites [13],[14],[19]. In addition, commensal bacteria are ingested by epithelial DCs, which subsequently migrate to MLNs for induction of T cell–independent, IgA B cell responses [20]. In summary, two broad types of S-IgA Abs reach our external secretions by transport across ECs and protect the epithelial surfaces from environmental insults, including infectious diseases.

It should be emphasized that several unique vaccine strategies are being developed to induce protective mucosal immunity. In this regard, delivery of mucosal vaccines by oral, nasal, or other mucosal routes requires specific adjuvants or delivery systems to initiate an immune response in MALT [21],[22]. However, a major benefit of mucosal vaccine delivery is the simultaneous induction of systemic immunity, including CD4^+^ Th1 and Th2, CD8^+^ cytotoxic T lymphocytes (CTLs), and Ab responses in the bloodstream, which are predominantly of the IgG isotype [21]. This, of course, provides a double layer of immunity in order to protect the host from microbial pathogens encountered by mucosal routes. This is especially promising for development of vaccines for developing countries, as well as those to protect our aging population [23],[24].

In this issue of *PLOS Biology*, Lau et al. used a multi-scale in vivo systems approach to assess how cells of the intestinal MIS communicate with intestinal ECs in response to an inflammatory signal [25]. The present study centered on the use of the proinflammatory cytokine tumor necrosis factor-alpha (TNF-α) given intravenously (i.v.) to assess its effects on the gut epithelium in the presence (wild-type [WT] mice) or absence (Rag1 knockout mice) of adaptive T and B lymphocytes. It is well known that TNF-α regulates many EC effects, including programmed cell death (apoptosis), survival, proliferation, cell cycle arrest, and terminal differentiation [26]. The authors had previously shown that TNF-α given i.v. to WT mice resulted in two different response patterns in the small intestine [27]. In the duodenum, which adjoins the stomach, TNF-α enhanced EC apoptosis, while in the ileum, the part next to the colon, an enhancement of EC division was seen [27]. In the present study, i.v. injection of TNF-α induced apoptosis in the duodenum (but not ileum) of WT, with heightened cell death in Rag1 mice [25]. Loss of either T or B lymphocytes also led to increased EC apoptosis, suggesting that both cell types are required to protect the epithelium from cell death. Also intriguing was the finding that eliminating the gut microbiota by antibiotic treatment did not affect the degree of EC apoptosis seen. Mathematical modeling allowed the group to show that TNF-α-induced apoptosis involved several steps in mice lacking functional T and B cells. Analysis of potential cytokines involved revealed that only a single chemokine, monocyte chemotactic protein-1 (MCP-1, C-C motif ligand 2 [CCL2]), protected ECs from apoptosis [25]. This new finding complements recent studies showing that IL-22, which is produced by several immune cells in the gut, plays a major role in protecting ECs from inflammation, infection, and tissue damage (Figure 3) [28].

**Figure 3:**
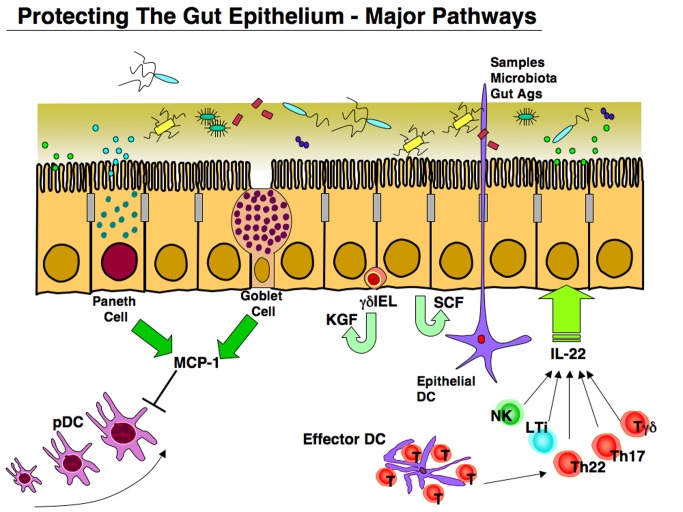
The gut epithelium exhibits several pathways that protect the integrity of this organ. Intestinal epithelial cells (ECs) produce stem cell factor (SCF), which induces proliferation and resistance to bacterial invasion. In addition, neighboring γδ intraepithelial lymphocytes (IELs) produce keratinocyte growth factor (KGF), which also stabilizes ECs. IL-22 produced by Th17, Th22, and γδ T cells as well as natural killer (NK) and lymphoid tissue inducer (LTi) cells plays a key role in both early and late phases of innate immunity in order to maintain the EC barrier. In addition, monocyte chemotactic protein (MCP-1) produced by Paneth cells and goblet cells down-regulates migration of plasmacytoid DCs (pDCs) into the intestinal lamina propria in order to decrease TNF-α-induced EC apoptosis.

Several unexpected discoveries followed. First, both goblet and Paneth cells were the major sources of MCP-1, and not the lymphoid cell populations that normally produce this chemokine (Figure 3). Second, the MCP-1 produced did not directly protect ECs, but instead acted via downregulation of plasmacytoid DCs (pDCs), a lymphocyte-like DC that produces various cytokines [29]. Finally, the study established that loss of adaptive (immune) lymphocytes resulted in decreased MCP-1 production, leading to increased pDC numbers and enhanced EC apoptosis. In the final experiment, the authors again showed that pDCs in the duodenum of Rag-1 mice produced increased levels of interferon-gamma that directly induced EC apoptosis. The message given by this intricate study is that systems biology approaches are quite useful in unraveling the complexities posed by the MIS in both health and disease.

The model developed by Lau et al. [25] could be useful to study several major problem areas. For example, a paucity of murine models exist to study food or milk allergies that usually affect the duodenum of the small intestine [30]. It is known that chemokine receptors control trafficking of Th2-type cells to the small intestine for IgE-dependent allergic diarrhea [31],[32]. The multi-scale systems approach could be used to assess much earlier responses to food or milk allergies in TNF-α-treated mice. A second avenue could well include the cell and molecular interactions that lead to intestinal EC damage resulting in IBD [33]. Clearly, progress is being made to study genetic aspects, regulatory T cells, and the contributions of the host microbiota to IBD development [17],[34]. Nevertheless, current mouse models have their “readout” as weight loss and chronic inflammation of the colon [17],[33],[34]. The Lau et al. approach could reveal cell-to-cell linkages that ultimately resulted in EC damage [25]. Further, this approach could reveal the earliest stages of pathogenesis of IBD before the influx of inflammatory cells causes the macroscopic changes characteristic of these diseases. Since the duodenum is normally sterile, one could have predicted their finding that antibiotic treatment to remove the gut microbiota would indeed be without effect. However, one wonders what effects would be seen in the stomach or in the colon, both of which can harbor a natural microbiota. Does TNF-α and antibiotic treatment alter the EC program in these mucosal tissue sites?

Finally, the intriguing question arising from the Lau et al. study [25] involves the finding that a full-blown adaptive immune system was required to maintain homeostasis and thus reduce EC apoptosis in the GI tract. Note that the response to in vivo TNF-α was assessed after only 4 hours, well before T and B cell responses could be manifested. How are early T and B cell signals transmitted to ECs? What are the mediators involved between the innate and adaptive components in the MIS for communication with the epithelium? As always, insightful studies raise many more questions than are answered. Nevertheless, the multi-scale in vivo systems analysis identified effects on the epithelium in a manner not appreciated up to now. The advantages of using an in vivo perturbation system is far superior to cell culture studies where only a few cell types are present.

## Abbreviations

Ab: antibody
Ag: antigen
AMP: anti-microbial peptide
APC: antigen-presenting cell
CTL: cytotoxic T lymphocyte
DC: dendritic cell
EC: epithelial cell
GALT: gut-associated lymphoid tissues
GI: gastrointestinal
IBD: inflammatory bowel disease
IEL: intraepithelial lymphocyte
i.v.: intravenously
M: microfold
MALT: mucosa-associated lymphoid tissues
MCP-1: monocyte chemotactic protein-1
MIS: mucosal immune system
MLN: mesenteric lymph node
NALT: nasopharyngeal-associated lymphoid tissues
pDC: plasmacytoid dendritic cell
pIgR: polymeric Ig receptor
RA: retinoic acid
S-IgA: secretory IgA
sIgA^+^: surface IgA-positive
TNF-α: tumor necrosis factor-alpha
UR: upper respiratory
WT: wild-type

## References

[1] Maslowski KM, Mackay CR (2011) Diet, gut microbiota and immune responses. Nat Immunol 12: 5–9. 21169997 10.1038/ni0111-5

[2] Kau AL, Ahern PP, Griffin NW, Goodman AL, Gordon JI (2011) Human nutrition, the gut microbiome and the immune system. Nature 474: 327–336. 21677749 10.1038/nature10213 PMC3298082

[3] Nicholson JK, Holmes E, Kinross J, Burcelin R, Gibson G, et al (2012) Host-gut microbiota metabolic interactions. Science 336: 1262–1267. 22674330 10.1126/science.1223813

[4] Hooper LV, Macpherson AJ (2010) Immune adaptations that maintain homeostasis with the intestinal microbiota. Nat Rev Immunol 10: 159–169. 20182457 10.1038/nri2710

[5] Garrett WS, Gordon JI, Glimcher LH (2010) Homeostasis and inflammation in the intestine. Cell 140: 859–870. 20303876 10.1016/j.cell.2010.01.023 PMC2845719

[6] Brenchley JM, Douek DC (2012) Microbial translocation across the GI tract. Annu Rev Immunol 30: 149–173. 22224779 10.1146/annurev-immunol-020711-075001 PMC3513328

[7] Ege MJ, Mayer M, Normand AC, Genuneit J, Cookson WO, et al (2011) Exposure to environmental microorganisms and childhood asthma. N Engl J Med 364: 701–709. 21345099 10.1056/NEJMoa1007302

[8] Sonnenberg GF, Monticelli LA, Alenghat T, Fung TC, Hutnick NA, et al (2012) Innate lymphoid cells promote anatomical containment of lymphoid-resident commensal bacteria. Science 336: 1321–1325. 22674331 10.1126/science.1222551 PMC3659421

[9] Hapfelmeier S, Lawson MA, Slack E, Kirundi JK, Stoel M, et al (2010) Reversible microbial colonization of germ-free mice reveals the dynamics of IgA immune responses. Science 328: 1705–1709. 20576892 10.1126/science.1188454 PMC3923373

[10] Nagler-Anderson C (2001) Man the barrier! Strategic defences in the intestinal mucosa. Nat Rev Immunol 1: 59–67. 11905815 10.1038/35095573

[11] Salzman NH, Hung K, Haribhai D, Chu H, Karlsson-Sjoberg J, et al (2010) Enteric defensins are essential regulators of intestinal microbial ecology. Nat Immunol 11: 76–83. 19855381 10.1038/ni.1825 PMC2795796

[12] Cheroutre H, Lambolez F, Mucida D (2011) The light and dark sides of intestinal intraepithelial lymphocytes. Nat Rev Immunol 11: 445–456. 21681197 10.1038/nri3007 PMC3140792

[13] Kiyono H, Kunisawa J, McGhee JR, Mestecky J (2008) The mucosal immune system. In: Paul WE, editor. Fundamental immunology. pp. 983–1030. Philadelphia: Lippincott Williams & Wilkins.

[14] Fujihashi K, Boyaka PN, McGhee JR (2008) Host defenses at mucosal surfaces. In: Rich RT, et al.., editors. Clinical immunology. pp 287–304. Philadelphia: Mosby Elsevier.

[15] Campbell DJ, Butcher EC (2002) Rapid acquisition of tissue-specific homing phenotypes by CD4^+^ T cells activated in cutaneous or mucosal lymphoid tissues. J Exp Med 195: 135–141. 11781372 10.1084/jem.20011502 PMC2196018

[16] Iwata M, Hirakiyama A, Eshima Y, Kagechika H, Kato C, et al (2004) Retinoic acid imprints gut-homing specificity on T cells. Immunity 21: 527–538. 15485630 10.1016/j.immuni.2004.08.011

[17] Izcue A, Coombes JL, Powrie F (2009) Regulatory lymphocytes and intestinal inflammation. Annu Rev Immunol 27: 313–338. 19302043 10.1146/annurev.immunol.021908.132657

[18] Brandtzaeg P (2007) Induction of secretory immunity and memory at mucosal surfaces. Vaccine 25: 5467–5484. 17227687 10.1016/j.vaccine.2006.12.001

[19] Brandtzaeg P (2010) Function of mucosa-associated lymphoid tissue in antibody formation. Immunol Invest 39: 303–355. 20450282 10.3109/08820131003680369

[20] Macpherson AJ, Uhr T (2004) Induction of protective IgA by intestinal dendritic cells carrying commensal bacteria. Science 303: 1662–1665. 15016999 10.1126/science.1091334

[21] Holmgren J, Czerkinsky C (2005) Mucosal immunity and vaccines. Nat Med 11: S45–S53. 15812489 10.1038/nm1213

[22] Wu Y, Wang X, Csencsits KL, Haddad A, Walters N, et al (2001) M cell-targeted DNA vaccination. Proc Natl Acad Sci U S A 98: 9318–9323. 11459939 10.1073/pnas.161204098 PMC55418

[23] Tokuhara D, Yuki Y, Nochi T, Kodama T, Mejima M, et al (2010) Secretory IgA-mediated protection against *V. cholerae* and heat-labile enterotoxin-producing enterotoxigenic *Escherichia coli* by rice-based vaccine. Proc Natl Acad Sci U S A 107: 8794–8799. 20421480 10.1073/pnas.0914121107 PMC2889329

[24] Fujihashi K, Kiyono H (2009) Mucosal immunosenescence: new developments and vaccines to control infectious diseases. Trends Immunol 30: 334–343. 19540811 10.1016/j.it.2009.04.004

[25] Lau KS, Cartez-Retamozo V, Philips SR, Pittel MJ, Lauffenburger DA, et al (2012) Multi-scale *in vivo* systems analysis reveals the influence of immune cells on TNF-α-induced apoptosis in the intestinal epithelium. PLoS Biol 10: e1001393 doi:10.1371/journal.pbio.1001393. 23055830 10.1371/journal.pbio.1001393 PMC3463506

[26] Schrofelbauer B, Hoffmann A (2011) How do pleiotropic kinase hubs mediate specific signaling by TNFR superfamily members? Immunol Rev 244: 29–43. 22017429 10.1111/j.1600-065X.2011.01060.x PMC3357464

[27] Lau KS, Juchheim AM, Cavaliere KR, Philips SR, Lauffenburger DA, et al (2011) In vivo systems analysis identifies spatial and temporal aspects of the modulation of TNF-alpha-induced apoptosis and proliferation by MAPKs. Sci Signal 4: ra16. 21427409 10.1126/scisignal.2001338 PMC3963028

[28] Sonnenberg GF, Fouser LA, Artis D (2011) Border patrol: regulation of immunity, inflammation and tissue homeostasis at barrier surfaces by IL-22. Nat Immunol 12: 383–390. 21502992 10.1038/ni.2025

[29] Reizis B, Bunin A, Ghosh HS, Lewis KL, Sisirak V (2011) Plasmacytoid dendritic cells: recent progress and open questions. Annu Rev Immunol 29: 163–183. 21219184 10.1146/annurev-immunol-031210-101345 PMC4160806

[30] Islam SA, Luster AD (2012) T cell homing to epithelial barriers in allergic disease. Nat Med 18: 705–715. 22561834 10.1038/nm.2760 PMC3863331

[31] Knight AK, Blazquez AB, Zhang S, Mayer L, Sampson HA, et al (2007) CD4 T cells activated in the mesenteric lymph node mediate gastrointestinal food allergy in mice. Am J Physiol Gastrointest Liver Physiol 293: G1234–G1243. 17916645 10.1152/ajpgi.00323.2007

[32] Blazquez AB, Knight AK, Getachew H, Bromberg JS, Lira SA, et al (2010) A functional role for CCR6 on proallergic T cells in the gastrointestinal tract. Gastroenterology 138: 275–284. 19782082 10.1053/j.gastro.2009.09.016 PMC2813342

[33] Saleh M, Elson CO (2011) Experimental inflammatory bowel disease: insights into the host-microbiota dialog. Immunity 34: 293–302. 21435584 10.1016/j.immuni.2011.03.008 PMC3108903

[34] Cho JH (2008) The genetics and immunopathogenesis of inflammatory bowel disease. Nat Rev Immunol 8: 458–466. 18500230 10.1038/nri2340

